# Beneficial effects of 6-shogaol on hyperglycemia, islet morphology and apoptosis in some tissues of streptozotocin-induced diabetic mice

**DOI:** 10.1186/s13098-019-0407-0

**Published:** 2019-02-12

**Authors:** Jun-Koo Yi, Zae-Young Ryoo, Jae-Jung Ha, Dong-Yep Oh, Myoung-Ok Kim, Sung-Hyun Kim

**Affiliations:** 1 Gyeongbuk Livestock Research Institute, Yeongju, 36052 South Korea; 2 School of Life Sciences and Biotechnology, Kyungpook National University, Daegu, 702-701 South Korea; 3 School of Animal BT Sciences, Kyungpook National University, Sangju, 742-711 South Korea; 4 China-US (Henan) Hormel Cancer Institute, No. 127 Dongming Road, Zhengzhou, 450008 Henan China

**Keywords:** 6-Shogaol, STZ, T1DM, ALT/AST, Hyperglycemia

## Abstract

**Background:**

Diabetes is characterized by hyperglycemia due to impaired insulin secretion and aberrant glucagon secretion resulting from changes in pancreatic islet cell function and/or mass. The aim of the present study was to investigate the effects of ginger on various tissues (i.e., pancreas, kidney, and liver) and insulin resistance in streptozotocin-induced diabetic mice. The pleasant aroma of ginger comes from the constituents present in its volatile oil, while its non-volatile pungent phytochemicals consist of gingerols, shogaols, and paradols.

**Methods:**

This research was conducted to determine the effects of 6-shogaol administration on blood glucose and insulin production in type 1 diabetic mice. Mice were intraperitoneally injected with shogaol at 5 or 10 mg/kg body weight. Untreated mice were injected with an equivalent volume of buffer, three times a week for 2 weeks. The animals were randomly divided into four experimental groups: control group mice (n = 3) were given an intraperitoneal (IP) injection of streptozotocin (STZ) vehicle (1 mL citrate buffer/100 g body weight) at day 1 and received an IP injection of 6-shogaol vehicle [1 mL buffer (0.5% DMSO, 10% Tween 20, and 89.5% PBS)/100 g body weight] every other day for 4 consecutive days.

**Results:**

6-Shogaol exhibited an antidiabetic effect by significantly decreased the level of blood glucose, body weight and attenuated the above pathological changes to the normal levels in the diabetic mice, and has effect against pancreas, kidney, liver damage in the diabetic mice. Since, 6-shogaol prevented the damage for STZ induced stress.

**Conclusion:**

6-Shogaol can be used as a therapeutic agent for preventing complications in diabetic patients. Diabetic treatment consider the 6-shogaol as a pharmatheuticals or combination drug with herbal plant or others 6-shogaol may be a good therapeutic drug because it covers not only pancreatic β-cell but also liver and kidney. Ginger may be ideal because they contain a variety of pharmacological compounds with different known pharmacological actions.

## Background

Diabetes mellitus (DM) is a common metabolic disorder, affecting 382 million people worldwide as of 2013 [1]. DM is characterized by high blood glucose levels due to impaired insulin action and secretion, and is classified into two major categories, types 1 and 2 [2]. Type 1 DM (T1DM) results from autoimmune destruction of β-cells in the pancreas [3], usually diagnosed in children and young adults, and was previously known as juvenile diabetes. Patients with T1DM must live in compliance with daily vigilance of blood glucose and insulin injections. Hyperglycemia is the hallmark of T1DM, inducing chronic generation of reactive oxygen species (ROS), consequently resulting in diabetic liver injury [4]. Patients with T1DM have a considerably worse long-term prognosis than individuals without diabetes, due to the high incidence of cardiovascular disease and end-stage renal disease (ESRD). Diabetic nephropathy (DN), the leading cause of chronic kidney disease in the United States, is responsible for up to 40% of all ESRD cases [5]. Since conventional and recently proposed therapies for DN lack major efficacy or are still under investigation, the search for novel targets involved in diabetes-induced renal damage is of primary importance.

Ginger is a commonly used spice or food supplement. This edible plant has been equally reputed for its medicinal function for centuries [6, 7]. The pleasant aroma of ginger comes from the constituents present in its volatile oil, while its non-volatile pungent phytochemicals, consisting of gingerols, shogaols, and paradols, give ginger its warm pungent sensation and are reported to account for most of its pharmacological effects [8, 9]. Among identified components, 6-gingerol was reported as the most abundant bioactive compound in ginger with various pharmacological effects, including antioxidant, analgesic, anti-inflammatory, and antipyretic properties [10–12]. Recent studies have shown that 6-shogaol, with the lowest concentration in ginger, is more biologically active than 6-gingerol [13–15]; it has also been reported as a potent anti-inflammatory and antioxidant compound [16].

In recent years, ginger has received extensive attention as a botanical dietary supplement in the United States and Europe because of its anti-inflammatory, anti-oxidative, and antitumor activities [17, 18]. A number of studies have examined the effects of ginger in hyperglycemia. Ginger (800 mg/kg) significantly decreased fasting blood glucose levels following 1-h treatment in an streptozotocin (STZ)-induced type 1 diabetic rat model [19] and prevented 5-hydroxytryptamine (5-HT)—induced acute hyperglycemia. Long-term treatment with ginger not only affected blood glucose levels, but also decreased serum triglyceride and total cholesterol, increased insulin, and effectively prevented liver and kidney damage in STZ-induced diabetic rats [20]. Of the several bioactive compounds identified in ginger, including gingerols, shogaols, paradols, and zingerones [21–23], 6-shogaol has recently been studied for its antioxidant and antitumor activities, as well as its activity in diclofenac sodium-induced liver injury [16, 24–26].

In the present study, we evaluated the effects of 6-shogaol on serum levels of blood glucose, body weight, and pathological changes in an STZ-induced mouse model. We also investigated the effect of 6-shogaol on cell proliferation and apoptosis in diabetic pancreas, kidney, and liver. We analyzed that 6-shogaol’s preventive effects of oxidative stress in STZ-induced mouse kidney, inhibitory effects of alanine transaminase (ALT) and aspirate aminotransferase (AST) levels, which are indicative of liver damage, and tumor necrosis factor (TNF)-α and transforming growth factor (TGF)-β1 mRNA expression levels in STZ-induced mouse liver. We verified that STZ-induced central areas of necrosis, fatty change, and inflamed liver sinusoids following treatment with 6-shogaol. We also analyzed expression levels of Ki-67 and other proteins related to cell proliferation in various tissues.

## Materials and methods

### Animal treatment

Male C57BL/6J (8-week-old) mice were purchased from Harlan Korea Laboratories. All mice were housed in the Experiment Animal Center of Kyungpook National University at 22 °C with a 12:12-h light/dark cycle and free access to rodent chow and tap water.

The animals were randomly divided into four experimental groups: control group mice (n = 3) were given an IP injection of STZ vehicle (1 mL citrate buffer/100 g body weight) at day 1 and received an IP injection of 6-shogaol vehicle [1 mL buffer (0.5% DMSO, 10% Tween 20, and 89.5% PBS)/100 g body weight] every other day for 4 consecutive days; STZ group mice (n = 3) were given a single IP injection of STZ (50 mg/kg body weight) at day 1, and a daily IP injection of sodium citrate vehicle for 4 consecutive days; Sho group mice (n = 4) received a single IP injection of cisplatin vehicle at day 0 (10 mg/kg body weight), and a daily IP injection of 6-shogaol for 4 days; and STZ + Sho group mice (n = 6) received a single IP injection of STZ at day 1 (50 mg/kg body weight), followed by daily IP injections of 6-shogaol solution (5 or 10 mg/kg body weight) for 4 days. 6-Shogaol (purity > 96%) was purchased from Chengdu Push Biotechnology (Cat No: PS1753). This experiments were continued check the glucose level and body weight until 2 weeks.

### Biochemical assays

Following STZ treatment, on the day of sacrifice, mice were euthanized by intraperitoneal injection of a cocktail of xylazine, tiletamine and zolazepam (Rompun, Bayer and Zoletil, Virbac). After achieving deep anaesthesia, the subjects were exsanguinated by cardiac puncture using a heparinized syringe. The collected blood samples were centrifuged at 5000 rpm for 10 min at 4 °C, and the separated sera were stored at − 80 °C until analysis. Serum samples were used for the biochemical analysis of ALT and AST levels. Measurements were performed using an auto-analyzer.

### Histopathological studies

For preparation of pancreas, kidney and liver tissues we had to CO_2_-euthanized all mice in the current study. Pancreas, kidney, and liver tissues were formalin-fixed after isolation, embedded in paraffin after dehydration (2 h in PBS, 2.5 h in 50% ethanol, 2.5 h in 70% ethanol, 2.5 h in 80% ethanol, overnight in 90% ethanol, 2.5 h in 95% ethanol, 2.5 h in 100% ethanol, 5 min in xylene twice, 30 min in xylene/paraffin, and 30 min in paraffin three times), and cut into 4-µm sections. The 4-µm thick sections were rehydrated (30 s in 100% ethanol, 30 s in 90% ethanol, 30 s in 80% ethanol, and 30 s in 70% ethanol), dried overnight, and stained with hematoxylin and eosin (3 min rinse in distilled water, 1 min 30 s stain in Gill’s hematoxylin V, 3 min rinse in running tap water, 30 s counterstain in eosin, and 5 s rinse in tap water), then dehydrated (10× dipping in 80% ethanol, 90% ethanol, 100% ethanol, and 100% ethanol, followed by 5 min each in xylene I, II, and III) [27].

### Immunohistochemistry studies

The immunohistochemistry studies were performed as previously described [27]. Pancreas, kidney, and liver tissue slices from the different experimental groups were immersed in 10% formalin at room temperature overnight. The tissues were then embedded in paraffin, and the paraffin sections were cut. After deparaffinization, some sections were used for routine hematoxylin–eosin staining, while others were incubated with blocking serum for 30 min followed by noncommercial rabbit polyclonal antibody against rat caspase-3 (1:300; Cell Signaling, #9661S) and Ki-67 (1:200; Abcam, AB92742) overnight at 4 °C. The sections were rinsed with Tris-buffered saline containing 1% Tween (TBST), then immediately incubated with horseradish peroxidase (HRP)-conjugated secondary antibody against rabbit immunoglobulin for 1 h. To detect HRP labeling, a peroxidase substrate solution with diaminobenzidine (0.05% diaminobenzidine in TBST with 0.05% H_2_O_2_) was used. The sections were counterstained with hematoxylin before examination under a light microscope.

### Real-time PCR

On the day of sacrifice, mice were euthanized by CO_2_ chamber. After opening the abdominal wall, the intestinal tract was removed and cut according to the different anatomic regions. The content of the fresh kidney and liver tissues was separately collected in microtubes and quickly frozen and homogenize in liquid nitrogen. Total RNA was prepared from frozen tissues using TRIzol Reagent (Thermo Fisher Scientific) according to the manufacturer’s instructions. cDNA was synthesized using a Veriti 96-Well Fast Thermal Cycler (5 min at 65 °C, 1 h at 42 °C, 5 min at 95 °C, followed by 4 °C overnight). Gene expression levels were determined by real-time PCR using the StepOnePlus Real-Time PCR System (Applied Biosystems) with Power SYBR Green PCR Master Mix (Applied Biosystems) and the following primers: nuclear factor E2-related factor (Nrf)-2 forward: 5′-CTC GCT GGA AAA AGA AGT GG-3′ and reverse: 5′-GGA GAG GAT GCT GCT GAA AG-3′; TNF-α forward: 5′-GCT GAG CTC AAA CCC TGG TA-3′ and reverse: 5′-CGG ACT CCG CAA AGT CTA AG-3′; TGF-β1 forward: 5′-TGA GTG GCT GTC TTT TGA CG-3′ and reverse: 5′-AGC CCT GTA TTC CGT CTC CT-3′; and β-actin forward: 5′-GCG CAA GTA CTC TGT GTG GA-3′ and reverse: 5′-ACA TCT GCT GGA AGG TGG AC-3′. Reactions were performed according to the manufacturer’s instructions and analyzed following geometric normalization.

### Statistical analysis

All quantitative results are expressed as mean ± standard deviation. Statistically significant differences were obtained using Student’s *t*-test or one-way analysis of variance. p < 0.05 was considered to indicate statistical significance.

## Results

### Effect of administration of 6-shogaol on the level of blood glucose and body weight of type 1 diabetic mice

**Fig. 1:**
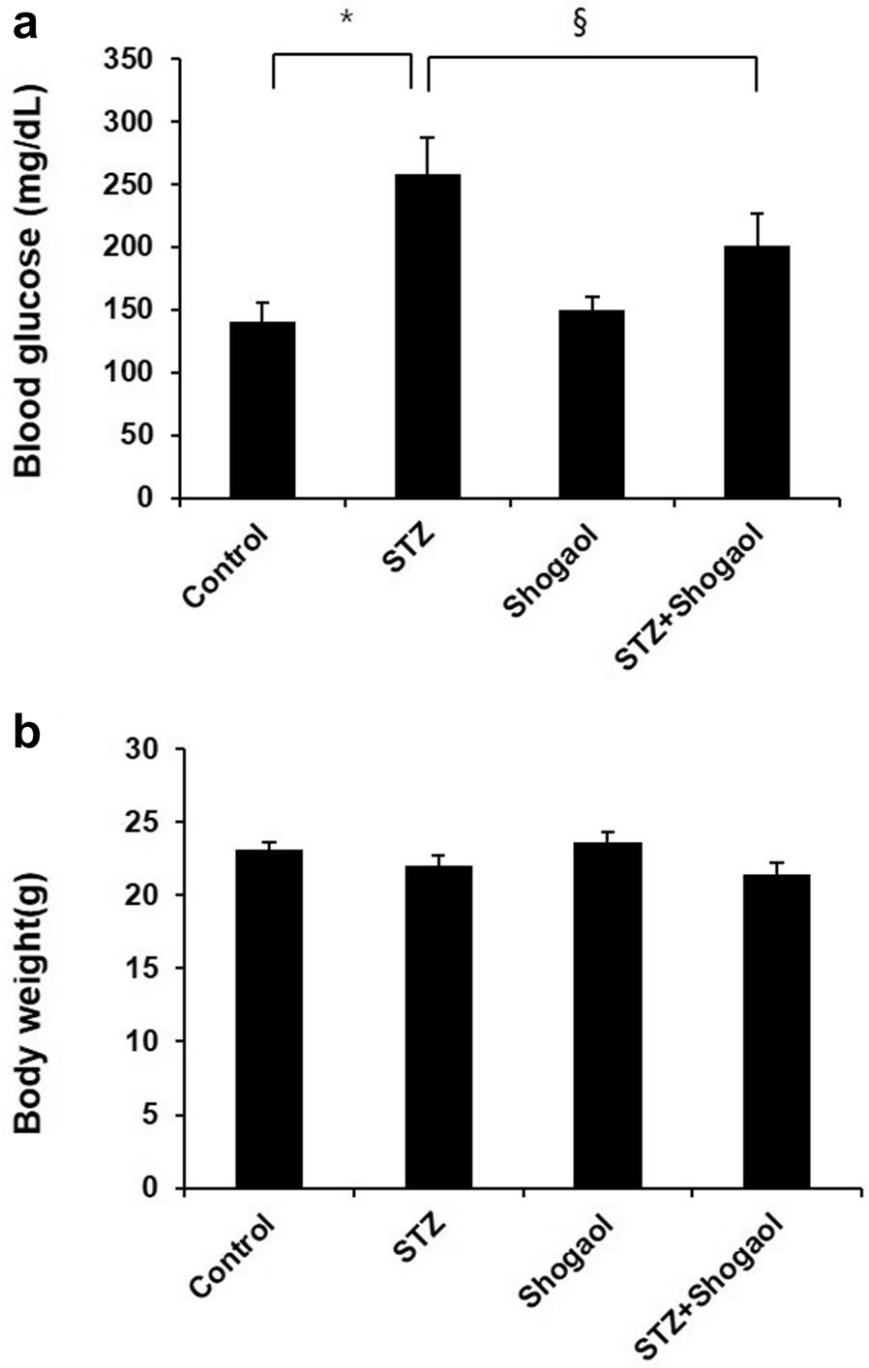
Effect of administration of 6-shogaol on the level of blood glucose and body weight of streptozotocin-induced type 1 diabetic mice. The type 1 diabetic mouse model was established by a STZ treatment for 2 weeks, which exhibited abnormal blood glucose tolerance (**a**) and body weight (**b**). The diabetic mice were treated with 6-shogaol at 10 mg/kg once every other day for the indicated time. Data are presented as means ± SEM. n = 4 in diabetic group and n = 3 in each other group. *p < 0.05 vs. the corresponding control group; ^§^p < 0.05 vs. the corresponding STZ group. Control, control mice; STZ, STZ induced diabetic mice without 6-shogaol treatment; Shogaol, control mice treated with 6-shogaol at 10 mg/kg; STZ + Shogaol, STZ induced diabetic mice with 6-shogaol at 10 mg/kg; STZ+Sho 10 mg/kg. (*p < 0.05 vs. Ctrl group, ^§^p < 0.05 vs. STZ group)

The type 1 diabetic mouse model was induced by streptozotocin showed a profound elevation in the level of blood glucose as compared to control group was noticed. Induction of the type 1 diabetic mouse model was evidenced by significant increases in blood glucose after 2 weeks. Treatment of the type 1 diabetic mice with low dose of 6-shogaol (10 mg/kg) for 2 weeks significantly decreased the level of blood glucose (Fig. 1a) (p < 0.05) but not body weight obviously restored after 2 weeks as compared with STZ group (Fig. 1b).

### 6-Shogaol prevented type 1 diabetes-induced pathological changes and immunohistochemistry in pancreas

**Fig. 2:**
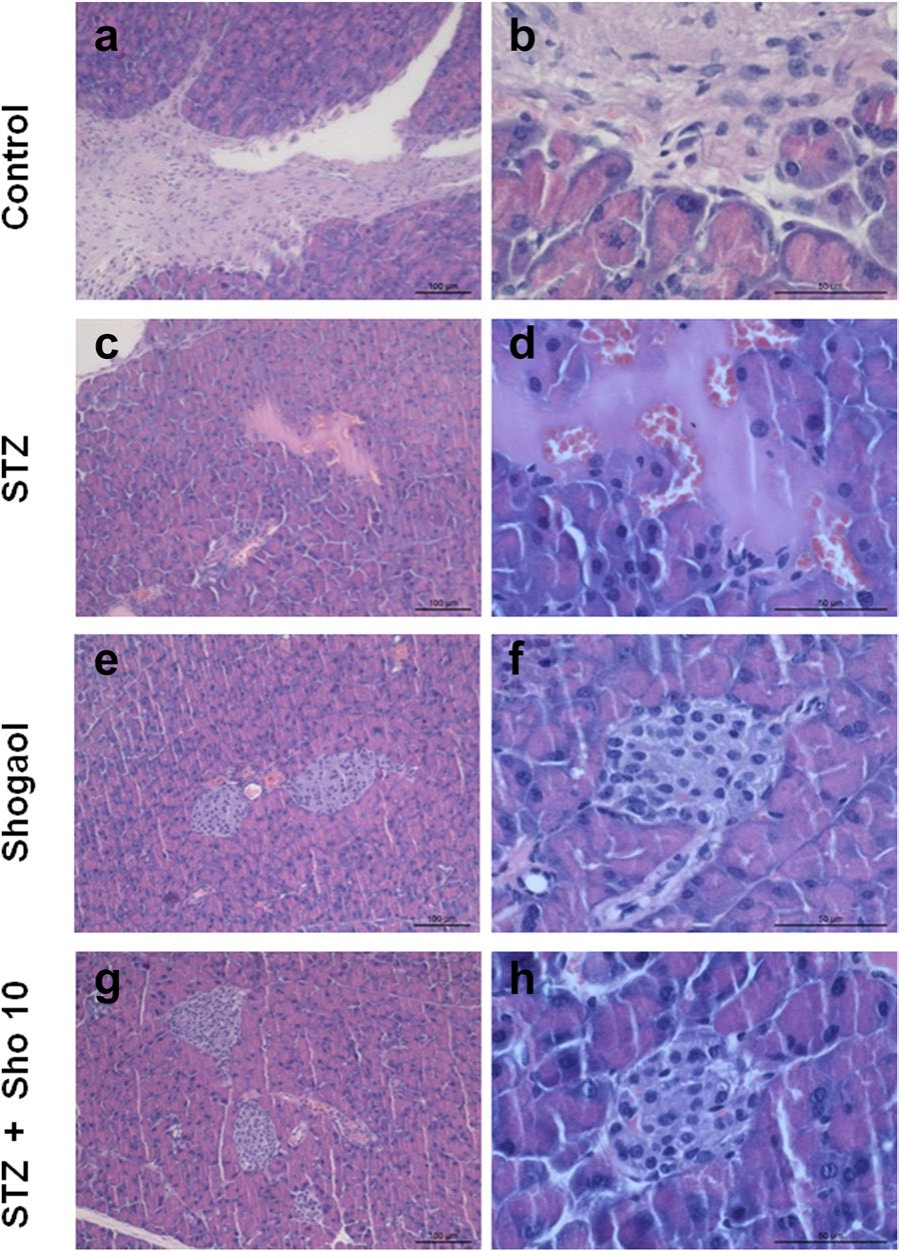
6-shogaol treatment changes on histopathological phenotype in the pancreas. Representative images of hematoxylin and eosin (H&E) staining for detection of renal pathological changes, Bowman’s capsule and glomerulus structure, respectively. Control group 100 μm (**a**), 50 μm (**b**), STZ-induced diabetic group 100 μm (**c**), 50 μm (**d**), 20 mg/kg of Shogaol treated group 100 μm (**e**), 50 μm (**f**), STZ-induced diabetic group with 10 mg/kg of Shogaol treated group 100 μm (**g**), 50 μm (**h**). 1.25 × and 100 × magnification. It was quantified using Image-Pro plus 6.0 software

Generally, pancreatic dysfunction reflects pathological changes in the diabetic pancreas. Compared with the control group (Fig. 2a, b), mice in the Con/sho at 10 mg/kg group displayed a normal structure of islets and ß-cell by H&E examination (Fig. 2e, f). However, the diabetic pancreas showed obvious abnormal islets structure and β-cell. Surprisingly, in many β-cells, large areas of cytoplasm were filled with a homogeneous unstructured substance that displaced the intracellular organelles (Fig. 2c, d). Treatment with 5 or 10 mg/kg group for 2 weeks markedly attenuated the above pathological changes (Fig. 2g, h).

**Fig. 3:**
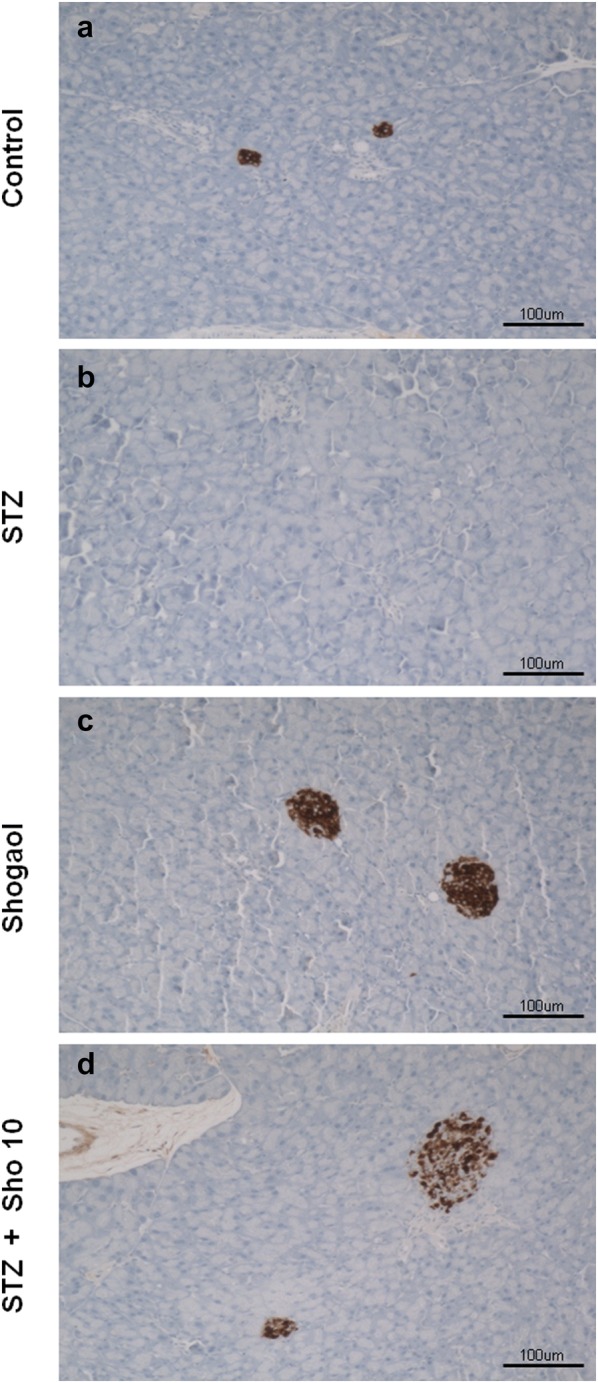
Immunohistochemistryfor insulin in pancreatic tissue from control (**a**), STZ (**b**), and Sho (**c**), STZ + Sho (**d**) mice after 2 weeks of treatment

STZ for 2 weeks led to marked changes in islet morphology. There was a dramatic decrease in insulin-positive cells compared with the STZ control group (Fig. 3b). Quantitative analysis revealed a marked reduction (~ 90%) in the area of the islet staining for insulin. 6-Shogaol therapy prevented the diabetes-induced changes in insulin (Fig. 3d) staining, and in the area of individual islets, or whole pancreas, composed of insulin-positive cells (Fig. 3a, c). 6-shogaol treatment was slightly more effective than insulin, perhaps because it produced more stable control of blood glucose (compare Fig. 1).

### Immunohistochemistry for caspase3 and ki-67 in pancreatic tissue from each experiment mice

**Fig. 4:**
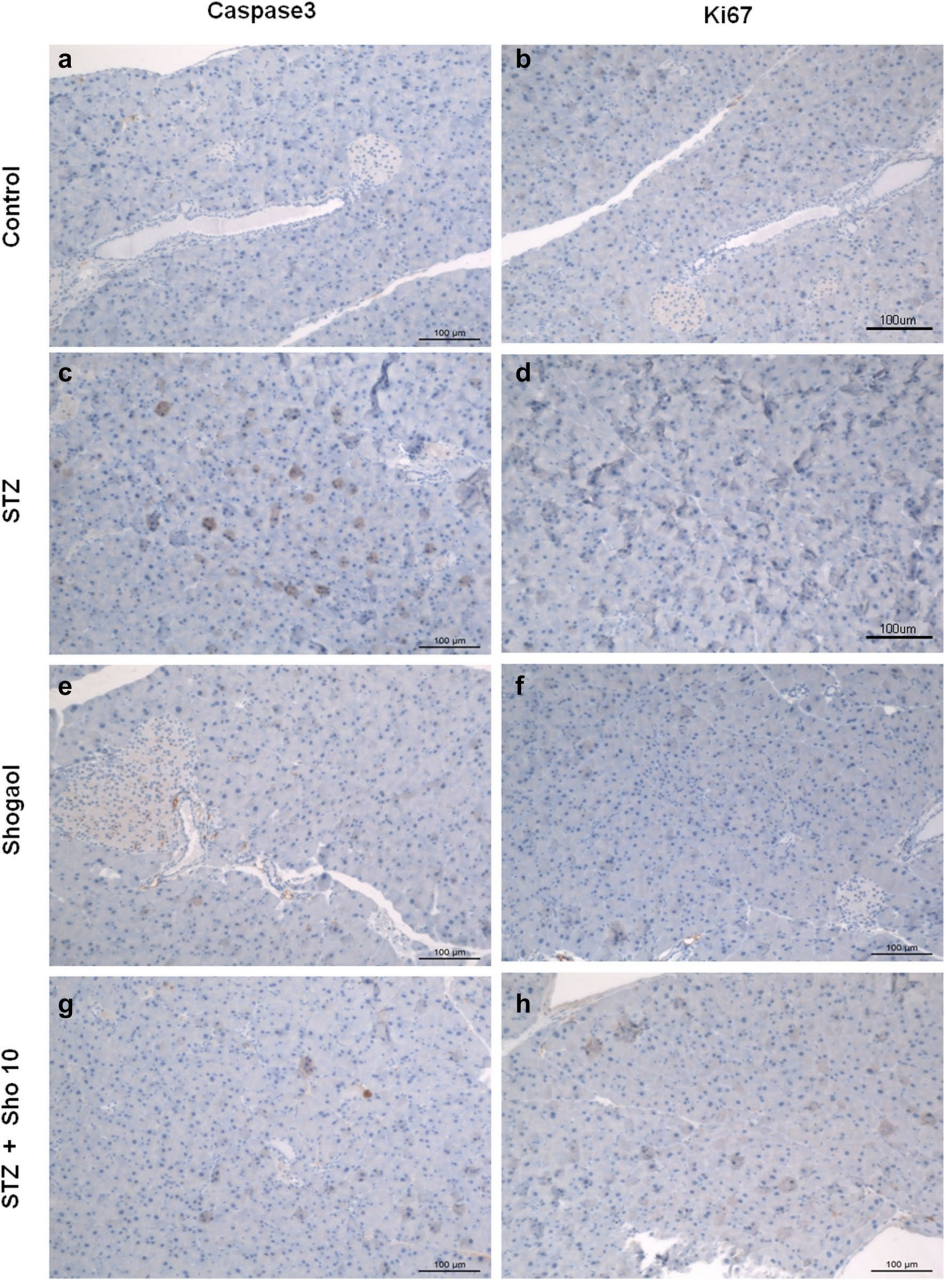
Immunohistochemistry for caspase3 and ki67 in pancreatic tissue from control (**a**), STZ (**b**), shogaol (**c**) and STZ + Sho (**d**) mice after 2 weeks of treatment. Immunohistochemistry for capase3 and ki67in renal from control (**a**, **b**), STZ (**c**, **d**), shogaol (**e**, **f**) and STZ + Sho (**g**, **h**) mice for 2 weeks. Serial sections from each mouse pancreas were stained using a noncommercial anti-capase3 and ki67 antibody. These pictures are representatives of typical samples obtained from four animals from each experimental group

To investigate the effect of 6-shogaol on cell proliferation and apoptosis in type 1 diabetic pancreas, we analyzed the immunohistochemistry of pancreatic tissue. Serial sections from each mouse pancreas were stained using a noncommercial anti-capase3 and ki67 antibody. Caspase3 labeling was associated with the execution-phase of cell apoptosis, and Ki67 labeling was associated with the cellular proliferation. These pictures are representatives of typical samples obtained from 4 animals from each experimental group. As shown in Fig. 4, caspase3 immunostaining study demonstrated that STZ + Sho group (Fig. 4g) was higher than in STZ group (Fig. 4c) whereas ki-67 positive cell no such a difference were recognized within and between the groups (Fig. 4b, d, f, h).

### 6-Shogaol prevented type 1 diabetes-induced pathological changes and immunohistochemistry in kidney

**Fig. 5:**
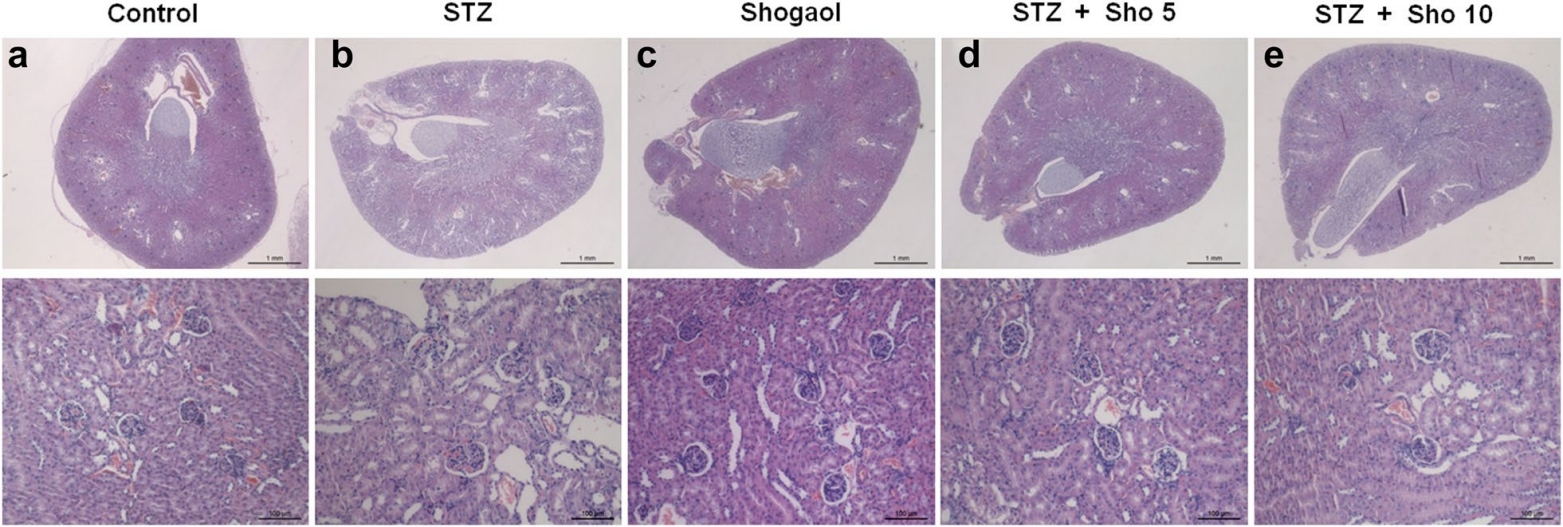
Effect of the administration of 6-shogaol on histopathological changes in the kidney of diabetic mice. Representative images of hematoxylin and eosin (H&E) staining for detection of renal pathological changes, Bowman’s capsule and glomerulus structure, respectively. ×1.25 and ×100 magnification. It was quantified using Image-Pro plus 6.0 software

Generally, renal dysfunction reflects pathological changes in the diabetic kidney. Compared with the control group, mice in the Con/sho at 10 mg/kg group displayed a normal structure of glomerulus and renal tubules by H&E examination (Fig. 5). However, the diabetic kidneys showed obvious Bowman’s capsule atrophy and abnormal glomerulus structure. Simultaneously, renal tubular dilation and epithelial cell degeneration were also observed in the diabetic kidneys. Furthermore, there were some bubbles in the renal tubules, which were attributed to excessive lipid accumulation in the diabetic kidneys (Fig. 5b). Treatment with 5 or 10 mg/kg group for 2 weeks markedly attenuated the above pathological changes (Fig. 5d, e).

### Immunohistochemistry for caspase3 and ki-67 in renal tissue from each experiment mice

**Fig. 6:**
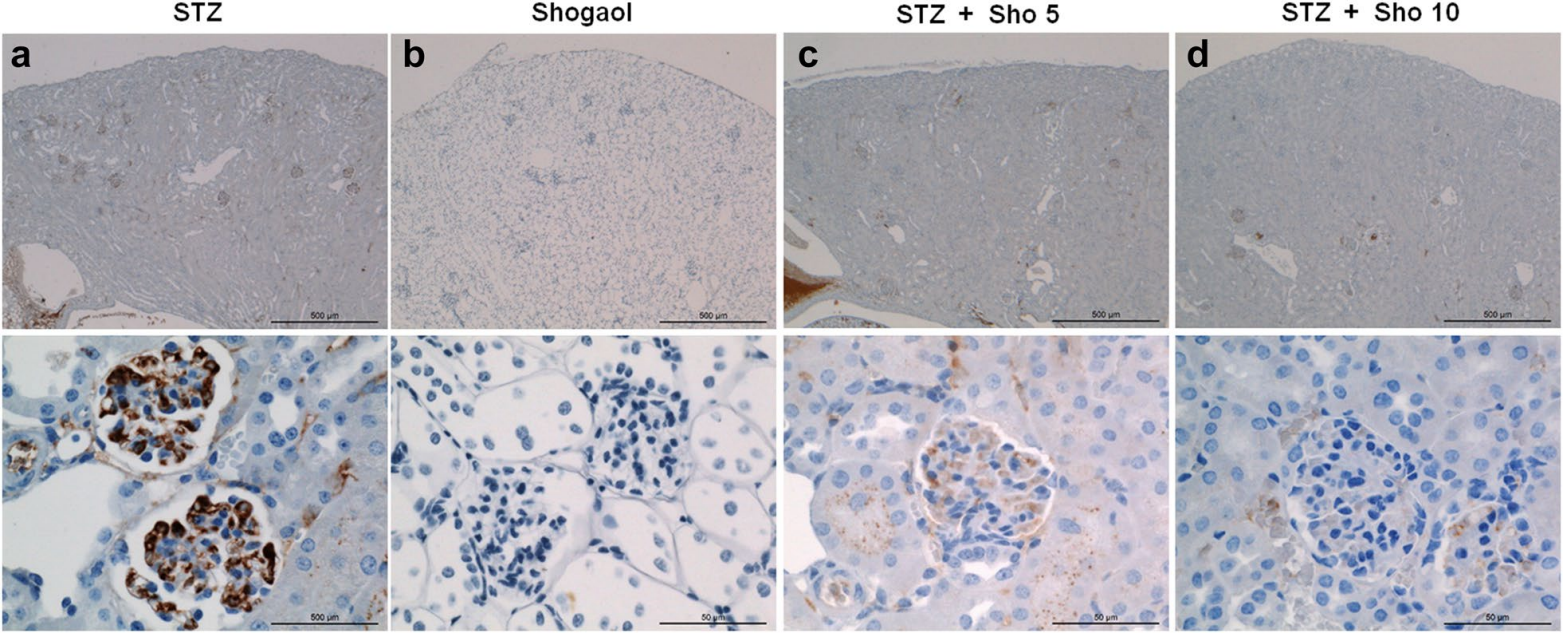
Immunohistochemistry for caspase3 in renal tissue from STZ (**a**), Sho (**b**), and STZ + Sho (**c**, **d**) mice after 2 weeks of treatment. Immunohistochemistry for capase3 in renal from STZ (**a**), Sho (**b**), STZ + Sho 5 mg/kg (**c**), and STZ + Sho 10 mg/kg (**d**) mice for 2 weeks. Serial sections from each mouse kidney were stained using a noncommercial anti-capase3 antibody. Capase3 labeling was associated with the execution-phase of cell apoptosis. These pictures are representatives of typical samples obtained from four animals from each experimental group

**Fig. 7:**
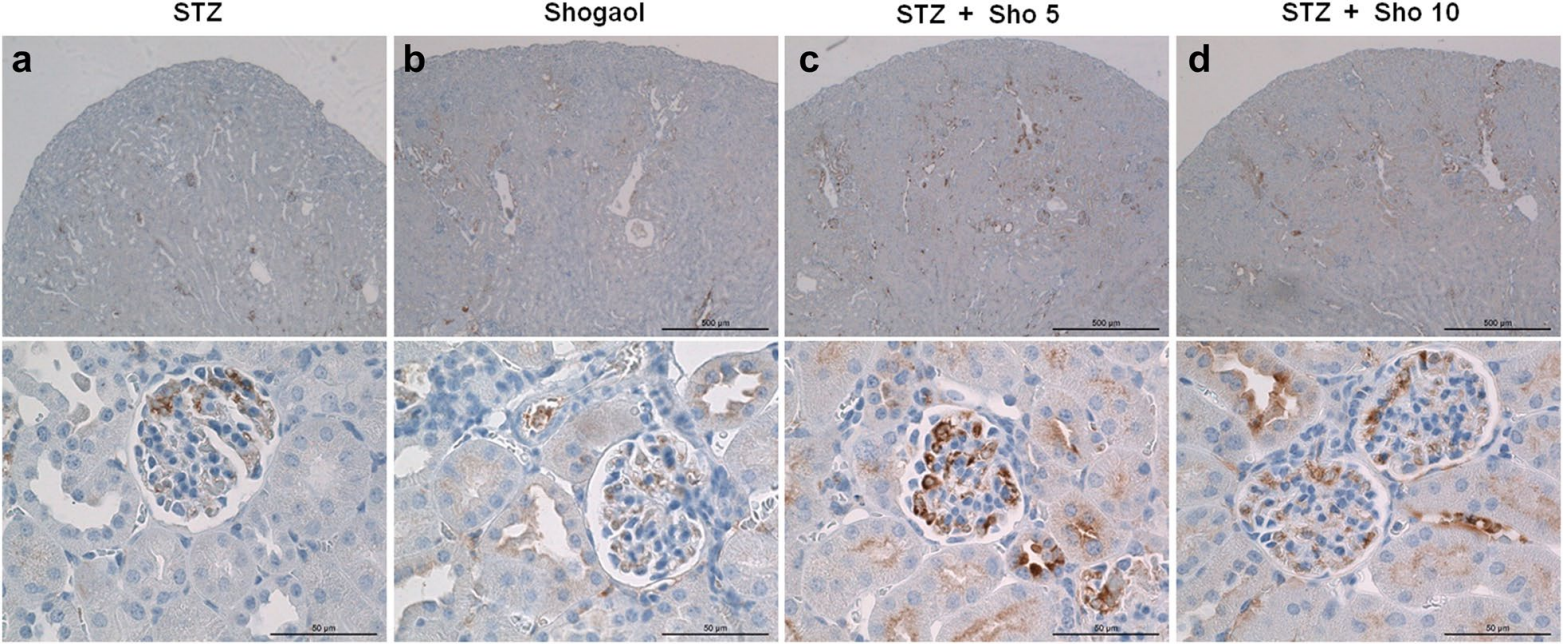
Immunohistochemistry for ki67 in renal tissue from STZ (**a**), Sho (**b**), and STZ + Sho (**c**, **d**) mice after 2 weeks of treatment. Immunohistochemistry for Ki67 in renal tissue from STZ (**a**), Sho (**b**), STZ + Sho 5 mg/kg (**c**), and STZ + Sho 10 mg/kg (**d**) mice for 2 weeks. Serial sections from each mouse kidney were stained using a noncommercial anti-ki67 antibody. Ki67 labeling was associated with the cellular proliferation. Furthermore it is associated with ribosomal RNA transcription. These pictures are representatives of typical samples obtained from four animals from each experimental group

Serial sections from each mouse kidney were stained using a noncommercial anti-capase3 and anti-ki67 antibody. Capase3 labeling was associated with the execution-phase of cell apoptosis. These pictures are representatives of typical samples obtained from 4 animals from each experimental group. Caspase3 immunostaining study demonstrated that STZ + Sho group was higher than in STZ group (Fig. 6). Whereas ki-67 positive cell no such a difference were recognized within and between the groups (Fig. 7). Ki67 labeling was associated with the cellular proliferation. Furthermore it is associated with ribosomal RNA transcription. These pictures are representatives of typical samples obtained from 4 animals from each experimental group. Caspase3 immunostainig study demonstrated that STZ + Sho group was higher than in STZ group (Fig. 6).

### 6-Shogaol prevented diabetic downregulation of renal nuclear factor E2-related factor-2 (Nrf-2) expression and function

**Fig. 8:**
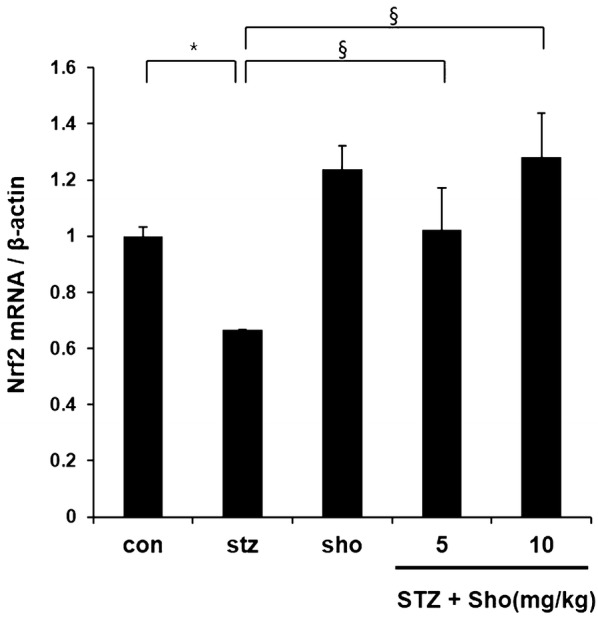
The effects of 6-shogaol on renal Nrf2 levels in type 2 diabetic mice. Renal tissues from different groups were collected at the indicated times for measuring Nrf-2 expression at the mRNA levels with RT-PCR, respectively. Data are presented as mean ± SEM. n = 4 in diabetic group and n = 3 in each other group. *p, 0.05 vs. the corresponding control group; ^#^p, 0.05 vs. the corresponding STZ group. Con, control mice; Sho 10 mg/kg, control mice treated with 6-shogaol at 10 mg/kg; STZ, STZ induced diabetic mice without 6-shogaol treatment; STZ + Sho 5 mg/kg, STZ induced diabetic mice with 6-shogaol at 5 mg/kg; STZ + Sho 10 mg/kg, STZ induced diabetic mice with 6-shogaol at 10 mg/kg

Nrf-2 is a key transcription factor that regulates intracellular redox balance and is a sensor of oxidative stress. Next, we determined whether 6-shogaol-induced renal protection against oxidative damage was associated with up-regulation of renal Nrf-2 levels. The results showed that the expression of Nrf-2 at the mRNA levels significantly decreased in the kidneys of diabetic mice. Multiple treatment of diabetic mice to 6-shogaol at 5 or 10 mg/kg almost completely prevented diabetic inhibition of renal Nrf-2 levels (Fig. 8). Since Nrf-2 is a transcription factor that positively regulates the expression of several downstream genes playing an important role in the prevention of oxidative stress and damage.

### 6-Shogaol prevented type 1 diabetes-induced pathological changes in liver

**Fig. 9:**
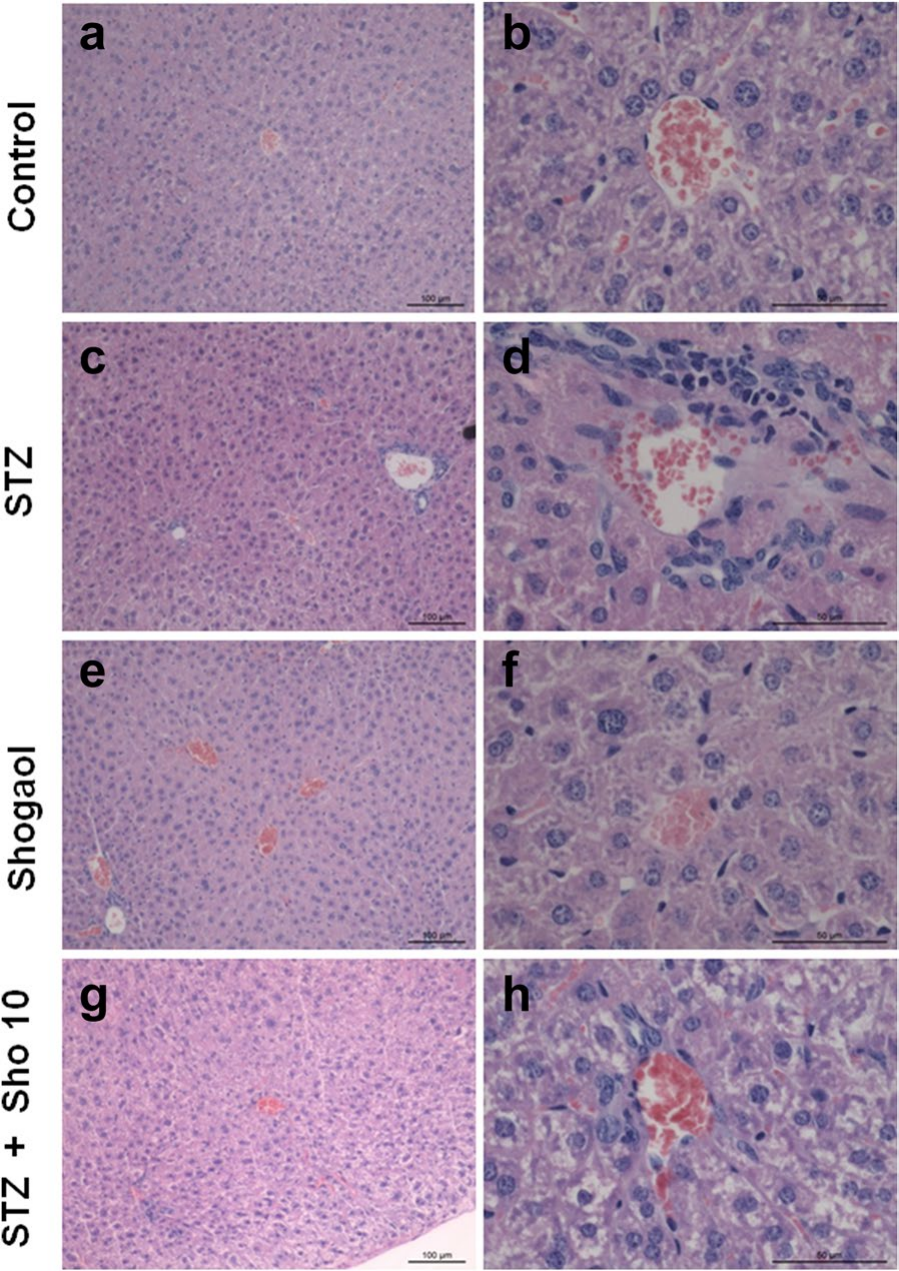
Effect of the administration of the 6-shogaol on the histopathology of the liver of type 1 diabetic mice (haematoxylin & eosin staining). Control group 100 μm (**a**), 50 μm (**b**), STZ-induced diabetic group 100 μm (**c**), 50 μm (**d**), 20 mg/kg of shogaol treated group 100 μm (**e**), 50 μm (**f**), STZ-induced diabetic group with 10 mg/kg of Shogaol treated group 100 μm (**g**), 50 μm (**h**)

To investigate the effect of 6-shogaol in type 1 diabetic liver, we analyzed the histology of liver tissue using hematoxylin and eosin staining (Fig. 9). The liver showed central area necrosis, fatty change and sinusoids with inflammatory cell in STZ-induced diabetic group (Fig. 9c, d), while nearly normal appearance of hepatic cells with some degree of swelling in STZ-induced diabetic group with 10 mg/kg of 6-shogaol treatment (Fig. 9g, h). 6-Shogaol treated group showed normal appearance of hepatic cells (Fig. 9e, f).

### Immunohistochemistry for caspase3 and ki67 in liver from each experiment mice

**Fig. 10:**
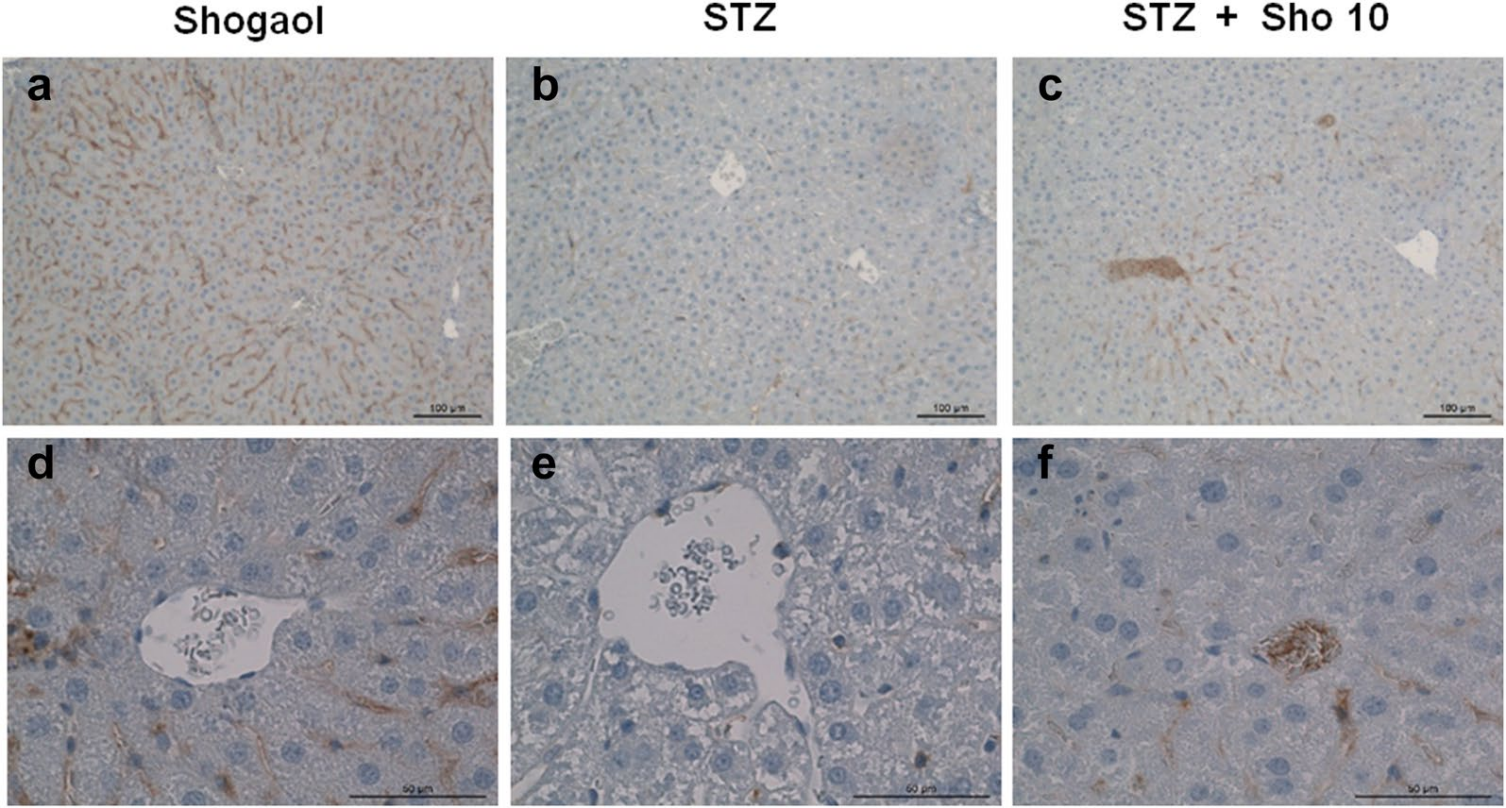
Ki-67 expression of the administration of the 6-shogaol on the the liver of type 1 diabetic mice. 6-shogaol treated group ×10 (**a**), STZ-induced diabetic mice ×10 (**b**), STZ-induced diabetic mice with 10 mg/kg of 6-shogaol treatment ×10 (**c**), 6-shogaol treated group ×40 (**d**), STZ-induced diabetic mice ×40 (**e**), STZ-induced diabetic mice with 10 mg/kg of 6-shogaol treatment ×40 (**f**)

**Fig. 11:**
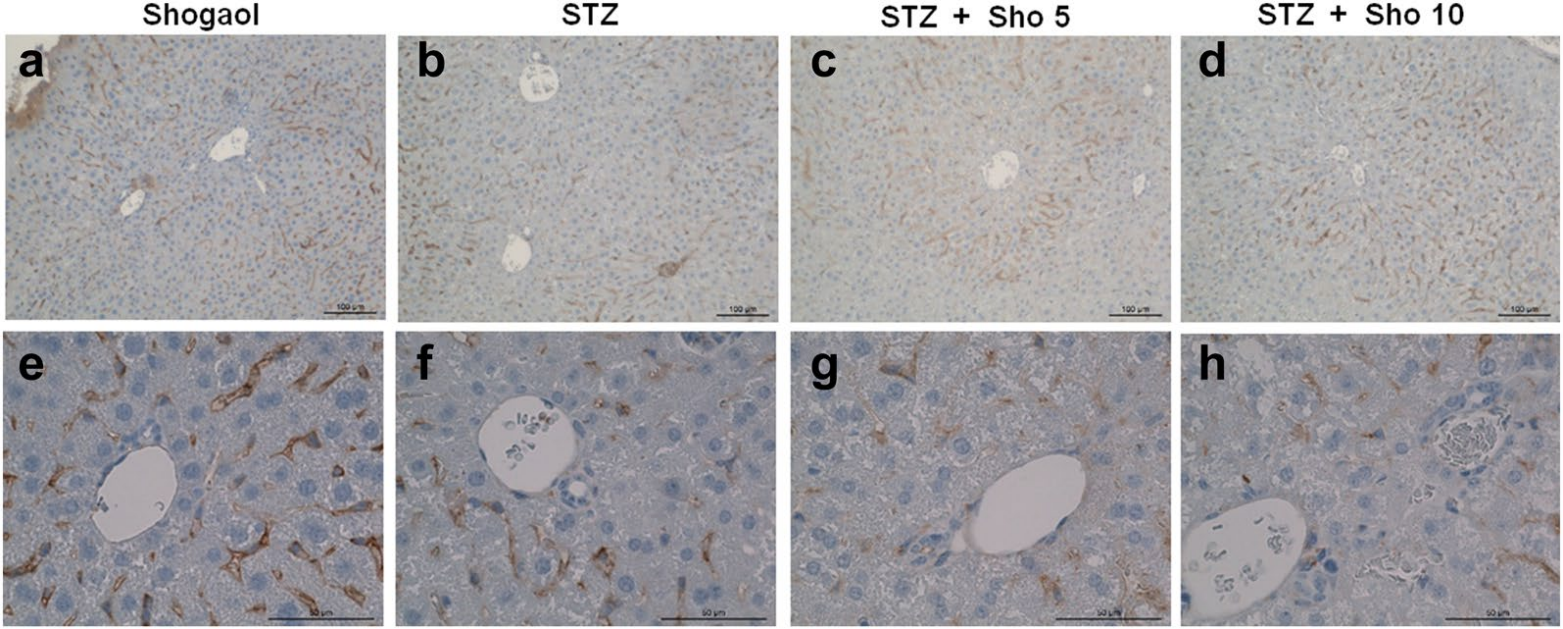
Caspase-3 expression of the administration of the 6-shogaol on the the liver of type 1 diabetic mice. 6-shogaol treated group ×10 (**a**), STZ-induced diabetic mice ×10 (**b**), STZ-induced diabetic mice with 5 mg/kg of 6-shogaol treatment ×10 (**c**), STZ-induced diabetic mice with 10 mg/kg of 6-shogaol treatment ×10 (**d**), [6]-shogaol treated group ×40 (**e**), STZ-induced diabetic mice ×40 (**f**), STZ-induced diabetic mice with 5 mg/kg of 6-shogaol treatment ×40 (**g**), STZ-induced diabetic mice with 10 mg/kg of [6]-shogaol treatment ×40 (**h**)

To investigate the effect of 6-shogaol on cell proliferation and apoptosis in type 1 diabetic liver, we analyzed the immunohistochemistry of liver tissue (Figs. 10, 11). ki-67 positive cell were decreased in STZ-induced diabetic mice (Fig. 10b, e). However, ki-67 positive cell were increased in 10 mg/kg of STZ + shogaol group (Fig. 10c, f). Caspase-3 was increased in STZ-induced diabetic mice (Fig. 10b, f), while nearly normal appearance of hepatic cells in both of STZ + shogaol group (Fig. 11c, d, g, h).

### Effect of 6-shogaol on serum activity of AST and ALT

**Fig. 12:**
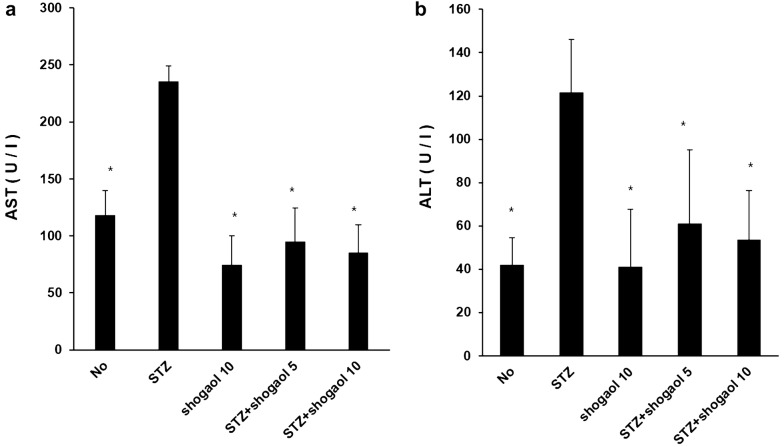
Effect of Shogaol on serum activity of AST and ALT in STZ-induced diabetic mice. *p < 0.05 in comparison with STZ-induced diabetic group

To investigate the effect of 6-shogaol in type 1 diabetic liver, we analyzed liver damage-related serum biochemical’s. In comparison with control group, ALT and AST levels were significantly increased in STZ-induced diabetic group. ALT and AST levels of STZ + 6-shogaol with 10 mg/kg group was significantly reduced in comparison with STZ-induced diabetic group. In STZ + 6-shogaol with 10 mg/kg group, ALT and AST levels were also reduced less than STZ + 6-shogaol with 5 mg/kg group (Fig. 12).

### Effect of 6-shogaol on level of RNA

**Fig. 13:**
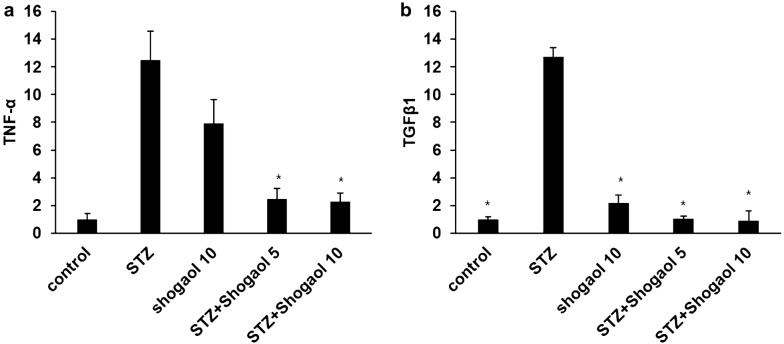
Effect of 10 mg/kg of shogaol on level of RNA in STZ-induced diabetic mice. *p < 0.05 in comparison with STZ-induced diabetic group

To investigate the effect of 6-shogaol on RNA levels, we analyzed the expression of inflammation-related genes in the liver, as demonstrated by quantitative real-time PCR analysis. In comparison with control group, TNF-α and TGFβ1 mRNA expression were significantly increased in the liver of STZ-induced diabetic mice. But, in STZ-induced diabetic group with 5 mg/kg and 10 mg/kg of 6-shogaol treatment, this were significantly reduced in comparison with STZ-induced diabetic group (Fig. 13).

## Discussion

Diabetic patient increased fastly in the world and this disorder increased heterogeneity, so more appropriate application need for therapy [28]. Recently, therapeutic drug has some drawbacks such as hepatotoxicity, vascular complications, neuronal and cardiotoxicity side effect. Chemotherapeutic drug still have a challenge for management of diabetes in medical system. This has led to an increase in the demand for natural products with anti-hyperglycemic activity and fewer side effects. Traditional plant medicines are used in the world wide for diabetic disease. The study of medicines field might offer a new key to unlock a diabetic pharmacy for the future.

Streptozotocin injection resulted in diabetes mellitus, which may be due to destruction of beta cells of Islets of Langerhans as proposed by others. Diabetes arises from irreversible destruction of pancreatic beta cells, causing degranulation and reduction of insulin secretion. STZ-induced diabetes is characterized by a severe loss in body weight and may exhibit most of the diabetic complications such as, myocardial, cardiovascular, nervous, kidney and urinary bladder dysfunction through oxidative stress [29]. After 30 days supplementation of ethanolic extract of ginger to diabetic rats, resulted significant diminution of fasting blood glucose level in respect to diabetic control rats, but no significant alteration of fasting blood glucose level to the control, which further strengthen the antidiabetogenic action of ginger extract. Many investigators reported that phenols, polyphenolic compounds and flavonoids of ginger are responsible for hypoglycemic and other pharmacological activities [30]. The decrease in body weight in diabetic rats shows that the loss or degradation of structural proteins is due to diabetes, and structural proteins are known to contribute to the body weight. The present study demonstrated that ginger treatment, ALT and AST levels were significantly increased in STZ-induced diabetic group. 6-Shogaol inhibits ALT (alanine transaminase), AST (aspirate aminotransferase) level which indicate liver damage and down regulates TNF-α, TGF-β1 mRNA expression in STZ-induced mice liver. And we identify STZ-induced central area necrosis, fatty change, sinusoids with inflammatory cell display nearly normal appearance by 6-shogaol treatment in various tissue. Also 6-shogaol decrease expression of ki-67, cell proliferation related protein, in addition proapoptotic protein caspase3 expression reverse normal condition level.

In our study, dramatic changes in insulin content, islet morphology and b-cell structure were evident after 2 weeks with STZ group compared with control group. There was a marked reduction in islet cells staining for insulin. Whereas, STZ + Sho group indicate that maintain of insulin immunolabelling and structure of b-cell mass after 2 weeks compared with control group. This is clear from the fact that many b-cells and insulin granules are observed at the structural level. The morphology of these b-cells also confirms they are undergoing apoptosis in various tissues. Nevertheless, ki-67 positive cell no such a difference were recognized within and between the groups in pancreas and kidney. We also found no change in proliferation. Thus, our data argue that the marked maintain in insulin staining we observe by immunohistochemistry is not primarily due to b-cell increase but rather to a increase in insulin gene expression and insulin granule density. The idea that increase insulin content can give rise to the fallacious impression of b-cell production has also been suggested for islets from patients with diabetes [31] and rodent models of diabetes [32].

DM is a global health problem due to its serious complications. Among diabetic complications, nephropathy probably is one of the major complications to increase the mortality of diabetic patients or impact their life quality. Although mechanisms by which diabetes induces the development of nephropathy are multiple, excessive production of reactive oxygen species (ROS) diabetic condition seems the primary factor [33–35]. Nuclear factor E2-related factor-2 (Nrf2) is a key transcription factor in regulating intracellular redox balance and a sensor of oxidative and electrophilic stress. Nrf2 regulates intracellular antioxidants, phase II detoxifying enzymes, and many other proteins that detoxify xenobiotics and neutralize ROS and/or RNS to maintain cellular redox homeostasis. NAD(P)H quinone oxidoreductase (NQO1), heme oxygenase-1 (HO-1), and glutathione S-transferase are among the well-studied Nrf2 target genes that are upregulated through the antioxidant response element regulatory element in response to oxidative stress [36–38]. The important role of Nrf2 in combating oxidative stress induced by diabetes has been demonstrated by the increased cardiac and renal sensitivity of Nrf2^−*/*−^ mice to diabetes [38–40].

Liver is a major target of insulin action, the onset of diabetes is accompanied by development biochemical and functional abnormalities in the liver including alteration in carbohydrate, lipid, protein metabolism, and change in antioxidant status [41–44]. In a research recently published, it is demonstrated that the diabetic state induces an increase of TNF-α and of its receptor TNF-R1 in the liver [45]. Also hyperglycemia induces apoptosis in streptozotocin (STZ)-induced diabetic rat liver through the increase of hydroxyl radical, and consequent activation caspase-3 [46]. STZ-induced animal models have been widely used in medical research to understand the pathophysiology of T1D based on the ability of STZ to disrupt pancreatic β-cells [47]. Aseer et al. [48] found that SPARC was significantly up-regulated in the liver while down-regulated in the pancreas of STZ-induced diabetic rats and significant up-regulation of TGF-β1, TNF-α in diabetic liver.

## Conclusion

Our study suggest that 6-shogaol has an effect against damage of pancreas, kidney, and liver in the diabetic mice. Since, 6-shogaol prevent the damage for STZ induced stress. 6-shogaol will applicate the diabetic therapy as a pharmatheuticals or combination drug with herbal plant or others. 6-shogaol may be a good therapeutic drug because it covers not only pancreatic β-cell but also liver and kidney. In the future Ginger may be ideal because they contain a variety of pharmacological compounds with different known pharmacological actions. However, further study will be needed, for the better understanding of the mechanism of action of ginger by which it modulates liver and kidney damage in diabetic condition.

## Abbreviations

IP: intraperitoneal
STZ: streptozotocin
DM: diabetes mellitus
T1DM: type 1 diabetes mellitus
ROS: reactive oxygen species
ESRD: end-stage renal disease
DN: diabetic nephropathy
5-HT: 5-hydroxytryptamine
ALT: alanine transaminase
AST: aspirate aminotransferase
TNF: tumor necrosis factor
TGF: transforming growth factor
HRP: horseradish peroxidase
Nrf: nuclear factor E2-related factor
NQO1: quinone oxidoreductase
HO-1: heme oxygenase-1
